# A Real-Life Study of Combined Treatment with Long-Term Non-Invasive Ventilation and High Flow Nasal Cannula in Patients with End-Stage Chronic Obstructive Lung Disease

**DOI:** 10.3390/jcm12134485

**Published:** 2023-07-04

**Authors:** Ulla Møller Weinreich, Line Hust Storgaard

**Affiliations:** 1 Department of Respiratory Diseases, Aalborg University Hospital, DK-9100 Aalborg, Denmark; line.storgaard@rn.dk; 2 Department of Clinical Medicine, Aalborg University, DK-9100 Aalborg, Denmark

**Keywords:** high-flow nasal cannula, HFNC, non-invasive ventilation, NIV, chronic obstructive pulmonary disease, COPD, palliative care

## Abstract

Patients with end-stage chronic obstructive pulmonary disease (COPD) often develop persistent hypoxic or hypercapnic respiratory failure, or a combination of both. Ventilatory support, in terms of a long-term high-flow nasal cannula (LT-HFNC) and long-term non-invasive ventilation (LT-NIV), may be indicated. Often, clinicians choose either one or the other. This paper explores combined treatment with LT-HFNC and LT-NIV in a real-life setting. In total, 33 patients with COPD and persistent respiratory failure were included in this study. Of those, 17 were initiated on LT-HFNC and used it for 595 (374) days and 16 were initiated on LT-NIV and used it for 558 (479) days. On average, patients used respiratory support continuously for 908 (586) days. Baseline characteristics were comparable, apart from PaCO_2_ at first ventilatory support initiation (LT-HFNC/LT-NIV 7.1 (1.1) kPa/8.8 (0.9) kPa respectively (*p* = 0.002)). Both groups experienced a reduction in hospitalizations in the first twelve months after treatment initiation, compared to the twelve months before (LT-HFNC *p* = 0.022 and LT-NIV *p* = 0.014). In total, 25% of LT-NIV patients stopped treatment after HFNC initiation due to intolerance and 59% stopped LT-HFNC treatment 126 (36) days after LT-NIV initiation as monotherapy was sufficient. In 44% of these patients, LT-HFNC was re-initiated at the end of life. At the time of analysis, 70% of patients had died. In the last three months of life, patients stopped using LT-NIV, whereas 91% used LT-HFNC. In conclusion, the combined use of LT-NIV and LT-HFNC reduced hospitalizations in patients with COPD and persistent respiratory failure. The study indicates that LT-HFNC is well tolerated, and better tolerated than LT-NIV at the very end stages of COPD.

## 1. Introduction

Approximately 400 million people suffer from chronic obstructive pulmonary disease (COPD) worldwide [1]. Patients with end-stage COPD may develop persistent respiratory failure. This may be either hypoxic or hypercapnic respiratory failure, or a combination of both. Persistent hypoxic failure is defined as when the partial pressure of oxygen (PaO_2_) in an arterial blood gas is <7.3 kilopascals (kPa) or <8.0 kPa in case of concomitant right-sided heart failure or a hematocrit > 55% during a clinically stable state [2]. The prevalence of persistent hypoxic failure in a COPD population is only described sparsely in the literature, but the prevalence in real-world cohorts is found to be between 2–16% [3,4]. Persistent hypercapnic failure is defined as a partial pressure of carbon dioxide (PaCO_2_) > 6 kPa, combined with elevated levels of standard bicarbonate (HCO_3_) > 26.9 mmol/L (males)/26.2 mmol/L (females), and base excess (BE) > 3 mmol/L in an arterial blood gas analysis, carried out whilst the patient is in a stable phase [5]. As with persistent hypoxic failure, the literature is sparse on the prevalence of persistent hypercapnic failure, but in European cohorts of patients with very severe COPD, a prevalence of 22–25% is seen [6,7]. The prevalence of combined persistent hypoxic and hypercapnic respiratory failure is not described in the literature.

Persistent hypoxic respiratory failure is associated with a reduction in health-related quality of life, exercise tolerance and skeletal muscle function, and increased mortality [8]. Persistent hypercapnic failure is an independent risk factor for COPD exacerbation, muscle wasting, comorbidities such as heart failure, diabetes, sleep apnea, and lastly, mortality [9,10,11,12].

Over the past decade, long-term non-invasive ventilation (NIV) has emerged and is now a recommended treatment for patients with persistent hypercapnic respiratory failure [2]. For patients with persistent hypoxic respiratory failure, long-term oxygen therapy (LTOT) has been the golden standard for four decades, reducing mortality [13]. However, long-term high-flow nasal cannula (HFNC) is an emerging add-on to LTOT treatment, reducing, for example, dyspnea and exacerbations of COPD [14]. Furthermore, qualitative data indicate that the treatment improves patients’ sleep and decreases the burden of COPD symptoms [15].

End-stage combined hypoxic and hypercapnic respiratory failure is an extensive disease burden to the patients, and it is therefore mandatory to identify treatment regimens that relieve patients’ symptoms.

The Danish guidelines for treatment with LT-NIV and LT-HFNC are accessible from the Danish Respiratory Society website. The guidelines for LT-NIV treatment were updated in 2022 [16], and LT-NIV is recommended at PaCO_2_ > 7.0 kPa two to four weeks after a hospital admission due to COPD exacerbation and three hospital admissions due to persistent hypercapnic respiratory failure within 12 months. LT-HFNC treatment is recommended for patients with persistent hypoxic failure and ≥2 exacerbations within 12 months; for patients with persistent hypercapnic failure, LT-HFNC may be considered if LT-NIV cannot be tolerated by the patient [17].

In this paper, we aim to deliver a real-life description of the combined use of NIV and HFNC in patients with end-stage COPD.

We aim to describe the patient population in which combined treatment is used, the reasons for treatment initiation, the order in which the two types of ventilatory support were started, the use of combined ventilatory support over time, and the effect on hospital admissions. Lastly, we aim to describe the use of the devices in the absolute terminal stages of the disease.

## 2. Methods

In this retrospective chart review, COPD patients treated with LTOT plus ventilatory support with either LT-HFNC or LT-NIV, in whom a second add-on ventilatory support (LT-NIV or LT-HFNC) was initiated in the period from 1 January 2017 to 31 December 2022, were included in this study. They were identified in the Aalborg University Hospital NIV and HFNC registries. All patients were followed and monitored through the Aalborg University Hospital ventilatory support outpatient clinic. Days of use of LT-NIV were registered through the digital platforms associated with the NIV equipment, (Airview^TM^, ResMed, San Diego, CA, USA and Care Orchestrator, Respironics Inc., © Koninklijke Philips, Amsterdam, The Netherlands) and days of use of LT-HFNC were registered through readings on the HFNC device (MyAirvo2^TM^, Fisher&Paykel Healthcare, Auckland, New Zealand) by the home supplier of the system (Vitalaire, Air Liquide, Paris, France).

Patients were prescribed LT-NIV according to the Danish Respiratory Society’s guidelines, as described above [16]. Prescription of LT-HFNC was also prescribed according to the Danish Respiratory Society’s guidelines as described above [18], however, the guidelines were not available until 2019. From 2017–2019, patients were prescribed LT-HFNC at the physicians’ discretion, however, the general practice did not differ from the above-stated. In addition to this, patients were to receive medical treatment for COPD according to national guidelines [19], including palliative care [20], the timeframe for the latter at the responsible physician’s discretion. Furthermore, if the responsible physician found an indication for physical training, patients were referred to this at their local municipality center, which cares for the training of patients with chronic diseases in Denmark.

When prescribed, patients, relatives, and other primary caregivers were instructed in the prescription, the use of the devices, the care for and cleaning of the devices, and the possibility of receiving backup from the outpatient clinic.

Patient hospital data were accessed through the national patient case file system. From the casefiles, the number of COPD-related hospital admissions during the past twelve months before the first ventilatory support initiation, the number of COPD-related hospital admissions during the past twelve months after the first ventilatory support initiation, as well as the duration of treatment with the first add-on device, were registered. The timepoint of the first initiation of a ventilatory support device was defined as the baseline. Baseline data were sex and age; duration of LTOT treatment; lung function, in terms of forced expiratory volume in the first second (FEV1) in liters and percent; Medical Research Council (MRC) score; body mass index (BMI); smoking status; and number and type of comorbidities. In addition, the date of death, of those no longer alive, was registered. Through the biochemistry data system, “the Clinical Laboratory Information Hospital System” (LABKA, Rigshospitalet, Copenhagen, Denmark), pH, PaCO_2_, PaO_2_, and HCO_3_ at the time of initiation of LT-HFNC and LT-NIV were registered. Furthermore, from the case file system, the reason for adding another ventilatory support device was registered. The data collection period was terminated on 5 May 2023.

For patients alive at the end of the study, their days in study were counted from initiation of the first respiratory support devices until 5 May 2023.

Data were registered in Research Electronic Data Capture (RedCap) (Vanderbilt University, Nashville, TN, USA). Results will be presented in percent of the total population, mean, and standard deviations. Comparison of subgroups was performed with paired *t*-test or chi^2^-test. Statistical analysis was performed with SPSS 27 (IBM, New York, NY, USA), and figures were made in Excel.

Permission to access patients’ case records was granted through the North Jutland Ethical Committee system (reg. nr 2023-009261) and the study was registered at the North Jutland Study database (reg.nr F2023-063). Patients still alive were contacted and gave informed consent to data access.

## 3. Results

In total, 42 patients were screened for this study. Out of those, 33 patients with COPD were included in the cohort, including ten who were alive at the end of the study. Reasons for failing the screening were treatment initiation outside the inclusion period (2), no overlap in treatment periods (5), and patients treated with respiratory support for reasons other than COPD (2). The baseline characteristics of the included patients are demonstrated in Table 1.

**Figure 1:**
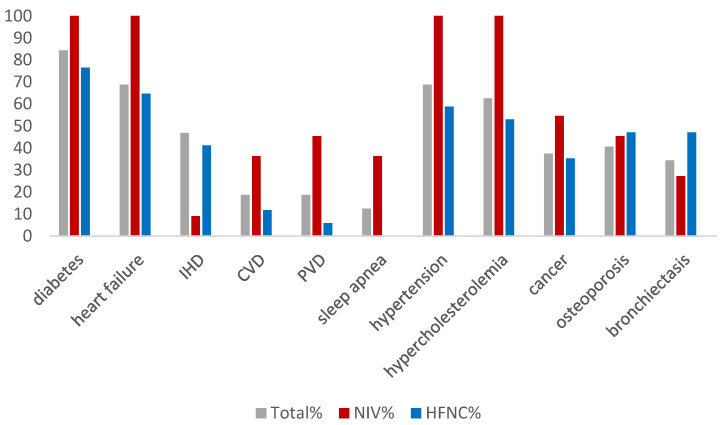
The percentage of patients suffering from comorbidities in the total population and within groups of patients initiated on ventilatory support with NIV (*n* = 16) or High Flow (*n* = 17). IHD = ischemic heart disease; CVD = cerebrovascular disease; PVD = peripheral vascular disease; Cancer = lung cancers, mammary cancers, prostatic cancers, gastrointestinal cancers, kidney cancer, bladder cancer.

On average, patients had 4.9 comorbidities. Figure 1 demonstrates the distribution of comorbidities in the total and sub-populations. Although there were no statistical differences, there was a tendency for patients who received LT-NIV as the first ventilatory support to have greater incidences of type 2 diabetes (*p* = 0.4), heart failure (*p* = 0.1), hypertension (*p* = 0.2), and hypercholesterolemia (*p* = 0.1).

In the study population, 51.5% (17/33) were treated with LT-HFNC and 48.5% (16/33) with LT-NIV as the first respiratory support device. Patients’ baseline characteristics in subgroups are described and compared in Table 2.

In both patients who were treated with LT-NIV and LT-HFNC as the first respiratory support, there was a significant reduction in the number of hospitalizations from 12 months before initiation compared to 12 months after initiation (*p* = 0.014 and *p* = 0.022, respectively).

On average, patients used respiratory support continuously for a total of 908 (586) days, during which they used LT-HFNC for 595 (374) days, LT-NIV for 558 (479) days, and used the two devices together for 289 (280) days. In total, 71% (12/17) of patients initially treated with LT-HFNC and 81% (13/16) of patients initially treated with LT-NIV started their second device within the first 12 months of treatment with ventilatory support. During the time period where patients combined the use of the two devices, patients were advised to use LT-NIV during the night and LT-HFNC during the daytime when at home. Furthermore, all patients were prescribed LTOT treatment, both at home and also as portable oxygen.

Table 3 shows the arterial blood gasses at the time of initiation of the second device for ventilatory support.

For patients who were initiated on LT-HFNC, an average increase in PaCO_2_ of 0.7 kPa (0.5) was seen at the time of LT-NIV initiation and an average increase in PaO_2_ of 0.5 kPa (0.3) was seen. For patients who were initiated on LT-NIV, an average reduction in PaCO_2_ of 0.5 kPa (0.4) was seen at the time of LT-HFNC initiation and an average reduction in PaO_2_ of 0.3 kPa (0.4) was seen.

**Figure 2:**
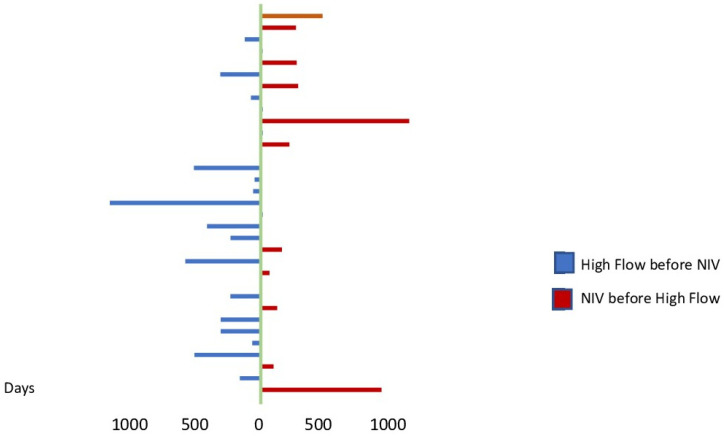
Days between the initiation of the two devices in the individual patients.

Figure 2 shows the time between the start of the first and the second respiratory support devices. 

**Figure 3:**
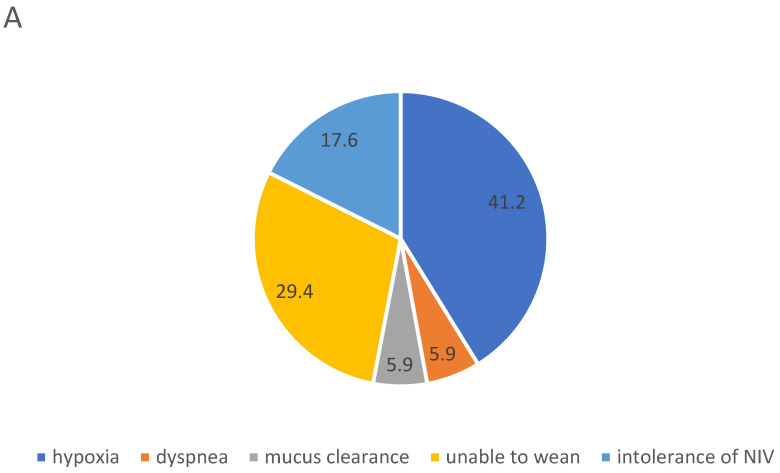

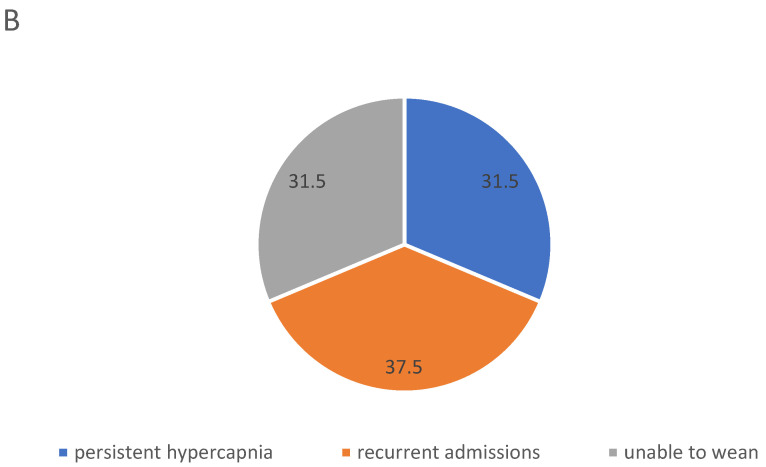
Primary reason for initiation of long-term high-flow Nasal Cannula (**A**) and long-term non-invasive ventilation (**B**) as second ventilatory support; in percent of number of patients in the respective subpopulations.

Figure 3A,B shows the reasons for the initiation of the second respiratory support device for LT-HFNC (A) and LT-NIV (B), respectively. In total, 53% (9/17) of patients who were initiated on LT-HFNC terminated the treatment on average 126 (36) days after starting LT-NIV treatment, all because LT-NIV also offered sufficient treatment for hypoxia. However, LT-HFNC was re-initiated in 44% (4/9) of these patients in the months prior to their death, to treat hypoxia and relieve dyspnea during hours without NIV or as a monotherapy. In total, 25% (4/16) of patients initiated on LT-NIV treatment stopped treatment on average 30 (8) days after starting treatment with LT-HFNC, all due to NIV intolerance.

Out of those alive at the end of the study, 90% (9/10) used both devices and one patient used LT-NIV. It is noteworthy that 70% (16/23) of patients died at home. Out of the remaining seven, 85% (6/7) died from acute respiratory failure, and one from aortic aneurysm bleeding.

**Figure 4:**
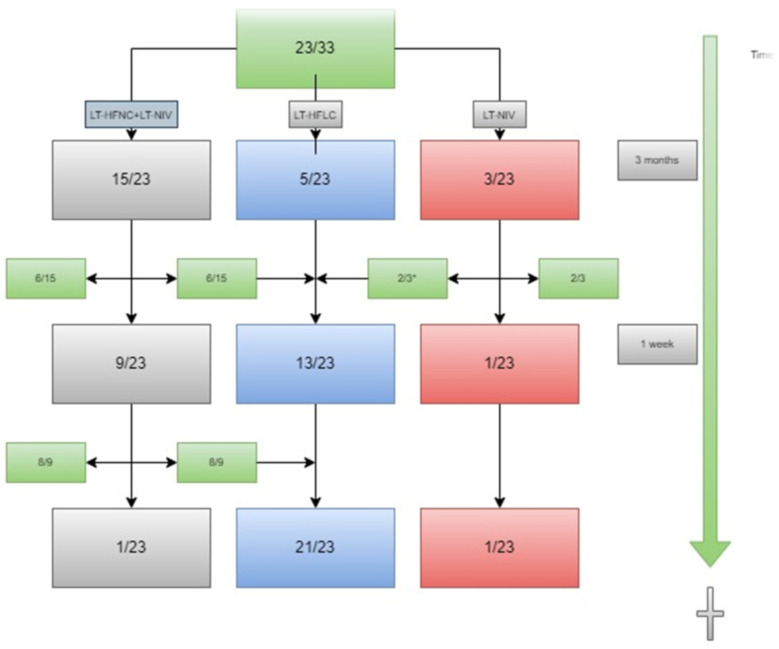
The use of ventilatory support devices within three months of the patients’ deaths. Data in absolute numbers. * LT-HFNC was re-initiated after LT-NIV was stopped. LT-HFNC = long-term high-flow nasal cannula; LT-NIV = long-term non-invasive ventilation.

In total, 70% (23/33) of patients died during the observational period. Figure 4 demonstrates the use of ventilatory support in the last three months of the patients’ lives. Two patients in the group of patients using two devices had re-initiated LT-HFNC shortly before this time. The figure shows that 11% (2/18) of the patients who used LT-NIV three months before they died used the device at their time of death. In total, 96% (22/23) of patients used LT-HFNC at the time of death.

**Figure 5:**
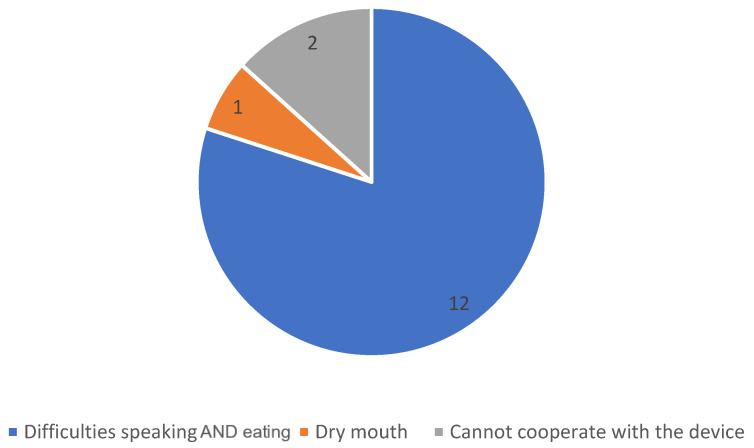
Reasons for terminating use of LT-NIV treatment. Data displayed in absolute numbers.

In 15 of the patients who terminated LT-NIV in their last months of life, the case files contained information about patients’ reasons for stopping the use of the device. The reasons are displayed in Figure 5. 

## 4. Discussion

This study explored combined LT-HFNC and LT-NIV treatment of COPD patients with persistent combined hypoxic and hypercapnic respiratory failure. The study showed that patients carried a high disease burden and, apart from those initially treated with LT-HFNC having a significantly lower PaCO_2_ than those initially treated with LT-NIV, patients were comparable at baseline. Furthermore, the study showed that a significant reduction in hospitalizations was seen in the year after treatment initiation, and that LT-HFNC was well tolerated during the patients’ last time alive.

The baseline characteristics of the patients in this study were similar, no matter which type of device was initiated as primary respiratory support; they had severely reduced lung function, were overweight, and had numerous comorbidities, not least heart failure and type 2 diabetes. In fact, patients who had LT-NIV as the first ventilatory support all had type 2 diabetes, heart failure, hypertension, and hypercholesterolemia, and although there were no statistical differences between the two groups, the cardiovascular comorbidities were more prevalent in patients initially treated with LT-NIV. Although both heart failure and diabetes are associated with hypercapnic respiratory failure [10,11,21], no studies on the prevalence of comorbidities in patients with COPD and persistent respiratory failure in long-term respiratory support treatment exist to these authors’ knowledge. Previous studies have found the prevalence of heart failure to be between 6–30% and type 2 diabetes between 9–12% in COPD patients [22,23]. In contrast, about 85% of patients in this cohort suffered from both heart failure and type 2 diabetes. It demonstrates that these patients carry a large disease burden, which may influence results.

In this study, PaCO_2_ differed between the two groups at initiation of first respiratory support, however, in both groups the average PaCO_2_ fulfilled the Danish National criteria for LT-NIV initiation [16]. In contrast to the Danish guidelines, the ERS guidelines do not specify at which level of hypercapnia LT-NIV should be considered, however, in the randomized controlled trial by Murphy et al., the inclusion criteria is PaCO_2_ > 7 kPa [24,25]. For LT-HFNC, the only existing guideline is the Danish Guideline, in which hypoxia in stable clinical conditions is the criteria for home treatment, however, persistent hypercapnic failure is not a contra-indication for treatment [26], although LT-NIV is considered the treatment of choice. Previous studies have, however, shown that LT-HFNC treatment is capable of stabilizing PaCO_2_ levels in COPD patients with persistent hypercapnic failure [27,28,29,30]. In a head-to-head comparison of LT-HFNC and LT-NIV in a 6-week cross-over study, the devices were equally effective in reducing PaCO_2_. This indicates that LT-HFNC could be a robust alternative to LT-NIV, not least for those who do not tolerate NIV treatment.

For both patients initiated on LT-HFNC and those initiated on LT-NIV, the number of COPD-related hospital admissions reduced significantly in the first twelve months after initiation of ventilatory support. This is in line with previous studies, which have shown the effect of both LT-NIV and LT-HFNC on hospital admissions [14,25,30]. Indeed, most patients started their second ventilatory support within this year, and a comparison of the effect of the two different devices on admissions is therefore not possible in this study. However, what these observational data suggest is, that in a real-life setting, combining LT-HFNC and LT-NIV treatment may be beneficial in patients with advanced COPD and persistent respiratory failure, in order to reduce hospitalizations.

Our data suggest that changes in arterial blood gasses have influenced the decision to prescribe a second ventilatory device to the patients. In patients initially treated with LT-HFNC, an increase in PaCO_2_ was seen at the time of NIV initiation, whereas in patients who were prescribed LT-NIV as the initial device, a reduction in PaO_2_ was seen at the time of LT-HNFC initiation. No clear recommendations exist at this time point, but our data suggest that blood gas changes should be included in future guidelines.

In this study, one-third of patients were initiated on LT-NIV due to the inability to wean patients from NIV treatment in the acute setting, in contrast to only 6% of LT-HFNC patients. In an international survey by Crimi et al., inability to wean was considered the second most important reason for LT-NIV treatment [31]. There are no data on LT-HFNC on this aspect. Inability to wean does, however, indicate a late initiation of ventilatory support. Frasier et al. have shown the beneficial effect of early initiation of LT-NIV on survival and healthcare costs in COPD. In future, we need to determine the right time for the initiation of ventilatory support, be it LT-HFNC or LT-NIV, to improve morbidity, mortality, and quality of life in patients with COPD.

Two-thirds of the patients included in this study had died at the time the analysis was carried out. This is unsurprising as ventilatory support is initiated for patients with persistent respiratory failure. However, little is known about how the use of the devices relieves symptoms, and how they are used by the patients in the very end stage of the disease. In a published protocol from 2021, Steindal et al. describes an intended scoping review of the effect of LT-NIV on symptoms in COPD palliative care, the outcome of which is not yet published [32]. Duarte et al. underline the lack of evidence on the effect of both LT-NIV and LT-HFNC in end-stage treatment of COPD patients, and emphasize the potential advantages of LT-HFNC as it may be used during eating and talking, both activities that may cause dyspnea in end-stage COPD [33]. This theory is supported by the findings of this study, as we found that patients’ primary reason for stopping the use of LT-NIV was incapability to speak and eat. Furthermore, in this study, the majority of patients used LT-HFNC until the time of death. However, future, possibly qualitative, studies need to be carried out to unfold the patient experiences of using ventilatory support in end-stage care.

In addition to this, it is noteworthy that the majority of patients died outside of the hospital. In general, it is the patients’ wish to die at home [34], but respiratory failure often makes it difficult to fulfill the patients’ wishes. Ventilatory support at the end of life may be able to help solve this issue.

This study has several limitations.

For one, it is a retrospective study based on clinical data, which has an effect on data quality. There are several parameters that would have been beneficial for this study, that were either not available or not registered at the study baseline, e.g., the hours of use of the devices were not included, as they are not available from the MyAirvo 2 device. Secondly, it is a single-center study, and the local set-up, know-how, and culture therefore determine treatment and thereby data. In addition to this, there are no guidelines for use, and treatment recommendations were therefore only at the prescribing physician’s discretion, and therefore not systematic. However, the aim has always been to improve patients’ quality of life, and therefore it has always been a recommendation to use LT-NIV during sleep and LT-HFNC during waking hours. This will, of course, affect the patterns of use and may explain why LT-HFNC seemed to be better tolerated during end-of-life care. Indeed, this paper is thought to be of inspiration to future study designs in an area of very limited scientific knowledge. Furthermore, the patients’ actual use of the devices goes beyond the healthcare personnel’s recommendations, and we need to learn from that for future study designs. Thirdly, patient-reported data such as symptom change on the type of support needed/used during the observational period could have been wished for. In the future, qualitative studies could be wished for to enlighten the subject. Lastly, a setup with two devices for ventilatory support may not be feasible in all respiratory clinics. Although studies have indicated that both LT-NIV and LT-HFNC treatments are cost-effective in patients with COPD and persistent respiratory failure [35,36,37,38], dual reimbursement may not be possible in all settings. Further evidence is therefore needed in this area to make combined use of the two devices possible.

## 5. Conclusions

In a real-life setting, the combined use of LT-NIV and LT-HFNC reduced hospitalizations in patients with COPD and persistent respiratory failure. Patients treated with two types of ventilatory support suffered from a considerable number of comorbidities. However, the study underlines our lack of knowledge about and consensus on both the potential target patient population and the indications for combined therapy. During the last three months of life, the vast majority of patients only used LT-HFNC. As such, the study indicates that LT-HFNC is well tolerated, and better tolerated than LT-NIV at the very end stage of COPD.

## Figures and Tables

**Table 1:** Baseline characteristics of the total study population, results are expressed as means (standard deviation) where nothing else is stated.

	Total Study Population
**Sex (%female)**	60.6
**Age (years)**	67.2 (7.1)
**MRC-score**	4.7 (0.3)
**FEV1, %**	28.6 (8.3)
**BMI (kg/m^2^)**	29.0 (5.8)
**Smoking status, present/former/never (%)**	33/67/0
**Time between initiation of long-term oxygen therapy and respiratory support (months)**	31 (12.7)
**Number of hospitalizations twelve months before initiation of first respiratory support**	2.1 (1.6)
**Number of hospitalizations twelve months after initiation of first respiratory support**	1.6 (0.4)
**PaO_2_ at baseline (kPa)**	7.4 (0.82)
**PaCO_2_ at baseline (kPa)**	7.9 (1.42)
**HCO_3_ at baseline (mmol/L)**	32.5 (5.0)

MRC = Medical Research Council; FEV1%: Forced expiratory volume in the first second in present of expected value (Global Lung Function Initiative); BMI = Body mass index; kPa = kilopascal; HCO_3_ = Standard Bicarbonate.

**Table 2:** Baseline characteristics of patients treated with LT-NIV or LT-HFNC, respectively. Results expressed as means (standard deviation) where nothing else is stated.

	LT-NIV (N = 16)	LT-HFNC (N = 17)	*p*-Value
**Sex, (%female)**	56.2	65.0	0.72
**Age (years)**	68.3 (7.5)	66.8 (7.3)	0.55
**MRC-score**	4.8 (0.2)	4.5 (0.5)	0.92
**FEV1%**	30.5 (8.8)	27.1 (8.2)	0.26
**BMI**	28.9 (6.5)	28.6 (5.5)	0.68
**Smoking status** **Present/former/never (%)**	25/75/0	35/65/0	0.23
**PaO_2_ at initiation (kPa)**	7.2 (0.9)	7.6 (2.1)	0.24
**PaCO_2_ at initiation(kPa)**	8.8 (0.9)	7.1 (1.3)	**0.002**
**St. Bicarbonate at initiation (mmol/L)**	34.4 (5.4)	30.7 (4.0)	0.10
**Number of hospitalizations twelve months before initiation of respiratory support**	2.9 (0.5)	2.5 (0.4)	0.13
**Number of hospitalizations twelve months after initiation of respiratory support**	1.6 (0.4)	1.5 (0.4)	0.84

MRC = Medical Research Council; FEV1%: Forced expiratory volume in the first second in present of expected value (Global Lung Function Initiative); BMI = Body mass index; kPa = kilopascal; HCO_3_ = Standard Bicarbonate.

**Table 3:** Arterial blood gas values at the time of initiation of the second device.

	LT-HFNC as Second Device	LT-NIV as Second Device
**pO_2_, kPa**	6.9 (1.6)	8.1 (1.1)
**pCO_2_, kPa**	8.2 (0.7)	7.8 (1.1)
**St. Bicarbonate, mmol/L**	31.4 (3.4)	32.1 (3.9)

LT-HFNC: Long-term high-flow nasal cannula; LT-NIV: Long-term non-invasive ventilation; kPa: kilopascal.

## Data Availability

The North Jutland Study database (reg.nr F2023-063).
